# Targeting the Divergent Roles of STK3 Inhibits Breast Cancer Cell Growth and Opposes Doxorubicin-Induced Cardiotoxicity In Vitro

**DOI:** 10.3390/cancers15102817

**Published:** 2023-05-18

**Authors:** Jiung Nam, Amelia U. Schirmer, Chelsea Loh, David H. Drewry, Everardo Macias

**Affiliations:** 1 Department of Pathology, Duke University School of Medicine, Durham, NC 27710, USA; 2 Structural Genomics Consortium and Division of Chemical Biology and Medicinal Chemistry, Eshelman School of Pharmacy, University of North Carolina at Chapel Hill, Chapel Hill, NC 27599, USA; 3 Lineberger Comprehensive Cancer Center, Eshelman School of Pharmacy, University of North Carolina at Chapel Hill, Chapel Hill, NC 27599, USA

**Keywords:** breast cancer, hippo kinase, yap, cardio-oncology, cardiotoxicity, STK3

## Abstract

**Simple Summary:**

Breast cancer is the second leading cause of cancer-related death in women. The increase in effective therapies and breast cancer survivorship has highlighted the cardiotoxic effects of many chemotherapies. In this study, we investigated Serine-Threonine Kinase 3 (STK3), a kinase in the Hippo Tumor-Suppressor Pathway, as a potential drug target to both inhibit breast cancer growth and mitigate chemotherapy-induced cardiotoxic damage. According to its canonical role, STK3 acts as a tumor suppressor, but STK3 is amplified and correlates with worse outcomes for breast cancer patients, suggesting that it plays a distinct role in breast cancer. Using genetic and pharmacological experiments in breast cancer and cardiomyocyte cell lines, we show that STK3 inhibition simultaneously slows breast cancer growth and invasion while providing protection to cardiomyocytes from doxorubicin-induced cardiotoxicity.

**Abstract:**

Breast cancer (BCa) is the most prevalent type of cancer in women. Several therapies used in the treatment of breast cancer are associated with clinically important rates of cardiovascular toxicity during or after treatment exposure, including anthracyclines. There is a need for new BCa therapeutics and treatments that mitigate chemotherapy-induced cardiotoxicity in BCa. In this study, we examine the effects of Serine/Threonine Kinase 3 (STK3) inhibition in the context of BCa therapy and cardioprotection from doxorubicin. STK3 (also known as MST2) is a key member of the Hippo Tumor-Suppressor Pathway, which regulates cell growth and proliferation by inhibiting YAP/TAZ co-transcription factors. Canonically, STK3 should restrict BCa growth; however, we observed that STK3 is amplified in BCa and associated with worse patient outcomes, suggesting a noncanonical pro-tumorigenic role. We found BCa cell lines have varying dependence on STK3. SUM52PE cells had the highest expression and dependence on STK3 in genetic and pharmacological assays. MCF-7 and MDA-MB-231 were less sensitive to STK3 targeting in standard proliferation assays, but were STK3 dependent in colony formation and matrigel invasion assays. In contrast, STK3 inhibition mitigated the toxic effects of doxorubicin in H9C2 rat cardiomyocytes by increasing YAP expression. Importantly, STK3 inhibition in BCa cells did not interfere with the therapeutic effects of doxorubicin. Our studies highlight STK3 is a potential molecular target for BCa with dual therapeutic effects: suppression of BCa growth and progression, and chemoprotection in cardiomyocytes.

## 1. Introduction

Breast cancer (BCa) is the second largest contributor to cancer-related deaths in women. Approximately 1 in 8 women in the United States will be diagnosed with BCa in their lifetimes, and 1 in 39 women will die from BCa [1,2]. The prevalence of BCa continues to rise globally due to increasing screening and shifting lifestyle trends [3,4]. The prognosis and treatment scheme of BCa is dependent on the presence of hormone receptors for estrogen (ER) and progesterone (PR) and the levels of human epidermal growth factor receptor two (HER2) [5]. Many treatments are aimed at targeting BCa through one of these receptors, such as hormone ER/PR modulators or targeted HER2 agonists. BCa lacking ER, PR, and HER2 is classified as “Triple-Negative” (TNBC) and does not respond to receptor modulator treatment [6]. For these patients, their treatment options are surgery, radiation, and chemotherapy. Research has been focused on molecular targeting in BCa, which addresses the need for increasing treatment options beyond the current endocrine therapies [5,7]. However, there remains a lack of actionable molecular targets to treat BCa, particularly TNBC.

Hippo signaling is a highly conserved tumor-suppressor pathway that regulates organ growth and proliferation [8,9]. The Hippo Pathway comprises Serine Threonine Kinases 3 and 4 (MST1/2), large tumor suppressors 1 and 2 (LATS1/2), Mob kinase activator (MOB1), and transcription cofactors Yes-Associated Protein 1 (YAP1) and TAZ [10,11]. Activation of YAP1 promotes cell proliferation and differentiation and has been implicated in tumor growth [10]. STK3/4 initiate a phosphorylation cascade that results in the downstream phosphorylation and degradation of YAP1. Therefore, the inhibition of STK3/4 is expected to increase YAP1 activity, which can have oncogenic effects in the context of cancer [9]. Thus, STK3/4 are canonically viewed as tumor suppressors. However, we and others have recently reported that STK3 has a pro-tumorigenic role in certain cancers that is distinct from its widely accepted role as a tumor suppressor [12,13,14,15]. In this regard, we recently reported that STK3, but not STK4, is amplified in prostate and BCa and expression is correlated with worse outcomes in patients with prostate cancer and BCa. We also reported that inhibition of STK3 genetically and with novel small-molecule inhibitors that we identified and validated slows prostate cancer growth and progression [13]. Taken together, these findings create a precedent that STK3 may also have a noncanonical tumor-promoting role in BCa and can be targeted to reduce BCa growth and proliferation.

Approximately 30% of BCa patients receive chemotherapeutic treatment based on anthracyclines, most commonly doxorubicin, daunorubicin, epirubicin, and idarubicin [16]. Long-term cardiovascular complications have become increasingly relevant to BCa patients as patient survivorship increases from improved cancer treatment strategies. The mechanism of this anthracycline-mediated cardiac damage comes from the production of reactive oxygen species (ROS) in cardiomyocytes and an elevation in oxidative damage [17]. To date, Dexrazoxane is the only drug approved by the US Food and Drug Administration to mitigate anthracycline-induced cardiomyopathy; however, it has not achieved widespread clinical use due to concerns that it may interfere with anthracycline activity in tumor cells [18]. Thus, there remains a need for a cardioprotective strategy that does not reduce the effectiveness of chemotherapy.

YAP1 activation has recently attracted interest as a mechanism for cardiac injury repair [9,19,20,21,22,23]. Activation of YAP1 in adult mice improves cardiomyocyte regeneration and contractility following myocardial infarction [24]. By contrast, activation of the Hippo Pathway (STK3/STK4 kinase activity) and the subsequent inhibition or degradation of YAP1 increases oxidative stress and cell death in cardiac cells [25]. Several studies have further shown that YAP1 activation counteracts doxorubicin-induced cardiomyopathy in vitro and ex vivo [26,27]. However, transcription factors such as YAP1 are notoriously difficult to modulate directly with small-molecule compounds [28]. By contrast, modulators for kinases, such as the upstream YAP1 regulators STK3/4, are routinely utilized in pharmaceutical discovery efforts [29]. Thus, targeted inhibition of STK3 may lead to stabilization and activation of YAP1 survival target genes that may counteract the effects of doxorubicin in cardiomyocytes.

In the current study, we explored the hypothesis that the inhibition of STK3 can have two clinically beneficial effects leading to potential overall improvement in BCa patient survival. First, in BCa, we tested if STK3 inhibition will slow BCa cell growth and invasion via its novel and noncanonical role. Secondly, in cardiomyocytes, we tested if STK3 inhibition and the consequent activation of YAP1 can improve the survival of cardiomyocytes post anthracycline treatment. In the present study, we employ pharmacological and genetic experiments to examine the effects of targeting the tissue-specific divergent roles of STK3.

## 2. Materials and Methods

### 2.1. Cell Culture

H9C2, MDA-MB-231, and MCF7 cells were grown in Dulbecco’s modified Eagle’s medium (DMEM) + 10% FBS. T47D cells were grown in RPMI 1640 + 10% FBS. SUM52PE cells were grown in 1:1 DMEM and Ham’s F-12 media mixture + HEPES, 5% FBS, 5 µg/mL insulin, and 1 µg/mL hydrocortisone.

### 2.2. Western Blots

Cells were lysed in a solution of radioimmunoprecipitation assay (RIPA) buffer, protease cocktail inhibitor 1, and phosphatase cocktail inhibitors 2 and 3 (MilliporeSigma, St. Louis, MO, USA). Protein content in the lysates were then quantified with detergent-compatible (DC) protein assay (Bio-Rad, Hercules, CA, USA), then buffered in Lamelli and RIPA. A total of 15–30 μg of protein was separated by SDS-PAGE gels, which were then transferred onto either 0.45 or 0.22 μm nitrocellulose membranes. The membranes were then developed with Pierce ECL Western (Thermo Scientific, Waltham, MA, USA #32106), SuperSignal West Pico PLUS Chemiluminiscent Substrate (#34580), SuperSignal West Dura Extended Duration Substrate (#34075), and SuperSignal West Femto (#3409). The following antibodies were used at a 1:1000 dilution: YAP (Cell Signaling Technologies (CST), Danvers, MA, USA #14074S), MST2 (Invitrogen Waltham, MA, USA #703027), p-MOB1 (#CST 8699s), p-P70 (#9204S), p42/49 (CST #4695S), Troponin (ProteinTech, San Diego, CA, USA, #15513-1-AP). GAPDH (CST #5174S) was used at a 1:4000 dilution. B-Actin (CST #5174S) was used at a 1:2000 dilution).

### 2.3. Proliferation and Drug Combination Assays

Standard 2D cell proliferation assays were conducted in 96-well plates. Cells were seeded in 100 μL of medium per well. Then, 24 h after seeding, wells were treated as specified for 72 h with 2× drug concentrations in 100 μL of medium. Total viable cell count was measured with Cell Counting Kit-8 (CCK8) (Cat# K1018Apex Bio, Houston, TX, USA). For drug combination assays, cells were pretreated with the STK3i compound for 24 h, then doxorubicin for 72 h. Total viable cell count was measured with CCK8. For colony formation assays, cells were plated at a 1000–5000 cell/well density in a 12-well plate in 1 mL of medium. After 2 weeks, the plates were fixed in 10% formalin and stained for visualization with 1% crystal violet solution. The plates were scanned and quantified with ImageJ for average area of colonies per well, normalized to the control. For spheroid assays, 500 cells were plated in a 384-well, U-shaped bottom plate and centrifuged and allowed to form spheroids overnight. After 24 h, cells were treated as specified. Scans were taken on a schedule using an Incucyte S3 live imaging system and quantified with the integrated image analysis software (Sartorius Inc, Ann Arbor, MI, USA).

### 2.4. Migration/Invasion Assays

Three-dimensional spheroid invasion assays were conducted in Primesurface U-bottom 96-well plates (S-BIO, Hudson, NH, USA). To allow spheres to form, cells were seeded in 50 μL of medium for 24 h. Matrigel, either with or without drug, was then added to each well at a concentration of 3 μg/mL and incubated for 1 h at 37 °C. An additional 100 μL of media with the specified treatment was then added to each well. Incucyte spheroid imaging was used to quantify total spheroid growth of the invasion front.

### 2.5. Lentiviral Infection

Stable constitutively active knockdown of STK3 was created with shRNAs in pLKO.1-puro vector backbone (MilliporeSigma, St. Louis, MO, USA). 293T cells were transfected with lentivirus containing psPAX2 and pMD2.G packaging plasmids using the Polyplus-transfection jetPRIME transfection kit. Virus was then collected after 48 h transfection. Virus-containing media and fresh media were added to target cells in a 1:1 ratio along with 8 μg/mL Polybrene infection reagent. After 24 h, cells were selected with 1–2 μg/mL puromycin until control cells were completely dead.

### 2.6. Statistical Analysis

Bliss synergy score was calculated using SynergyFinder 2.0 software and plotted. Synergy score tables were downloaded and replotted on GraphPad Prism Version 9.5.1 [30]. The synergy scoring criteria between two drugs is the following: <−10, the interaction is antagonistic; from −10 to 10, the interaction is additive; >10, the interaction between two drugs is synergistic. Cell viability and proliferation assays were analyzed by one-way ANOVA with Dunnett’s multiple comparisons test. Longitudinal spheroid growth or invasion studies were analyzed using 2-way repeated measures ANOVA with Dunnett’s multiple comparisons test.

## 3. Results

**Figure 1:**
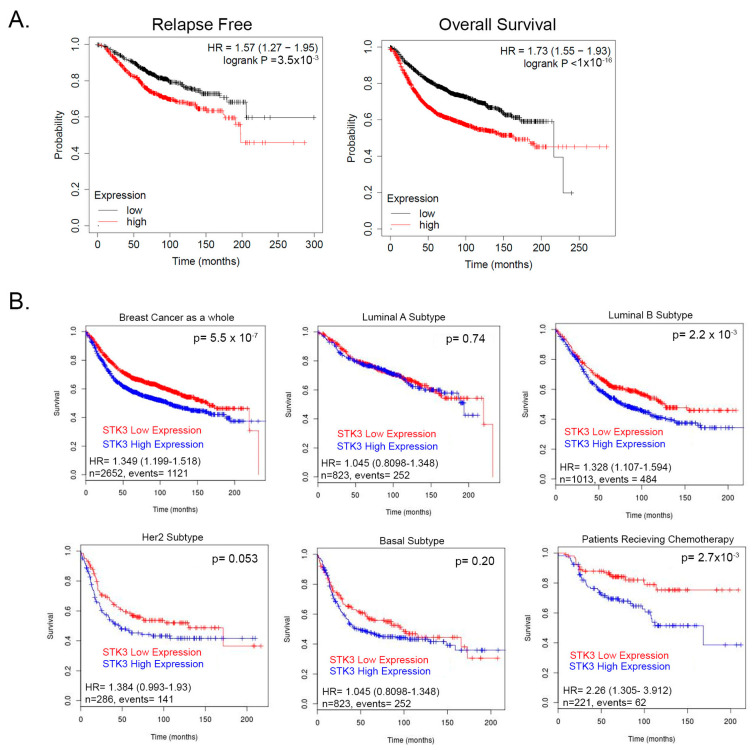
(**A**) Relapse and overall survival of BCa patients stratified by STK3 median upper and lower expression KM-plotter database. (**B**) Survival analysis of STK3 stratified patients by BCa subtypes and in patients receiving chemotherapy from pooled cohorts on BreastMark database.

We previously showed that contrary to its accepted role as a tumor suppressor, the STK3 gene is amplified in several cancer types and correlates with worse outcomes [13]. To further evaluate this observation, we queried a pooled BCa cohort of 7830 unique samples from 55 independent datasets in KM-plotter [31,32]. In this BCa dataset, STK3 high expression significantly correlated with decreased relapse-free (Figure 1A, HR = 1.57, 95% CI 1.27–1.95, *p* = 3.5 × 10^−5^, Log-rank) and overall survival (Figure 1A, HR = 1.57, 95% CI 1.27–1.95 *p* < 1 × 10^−16^, Log-rank). We then queried the BreastMark database to assess STK3 by BCa subtypes. In this BCa dataset as a whole, STK3 high expression correlated with worse overall survival (Figure 1B, HR = 1.349, 95% CI 1.199–1.518, *p* = 5.551 × 10^−7^). In luminal A, basal, and HER2 subtypes, there was no difference in survival with STK3 expression. However, in the luminal B subtype (HR = 1.328, 95% CI 1.107–1.594, *p* = 0.0022) and interestingly in patients receiving chemotherapy (HR = 2.26, 95% CI 1.305–3.912, *p* = 0.0028), high expression of STK3 significantly decreased overall survival. These clinical correlative data suggest that STK3 plays a noncanonical role in BCa and may be more important in different subtypes.

### 3.1. SUM52PE BCa Cells Are an STK3-Dependent Cell Line

**Figure 2:**
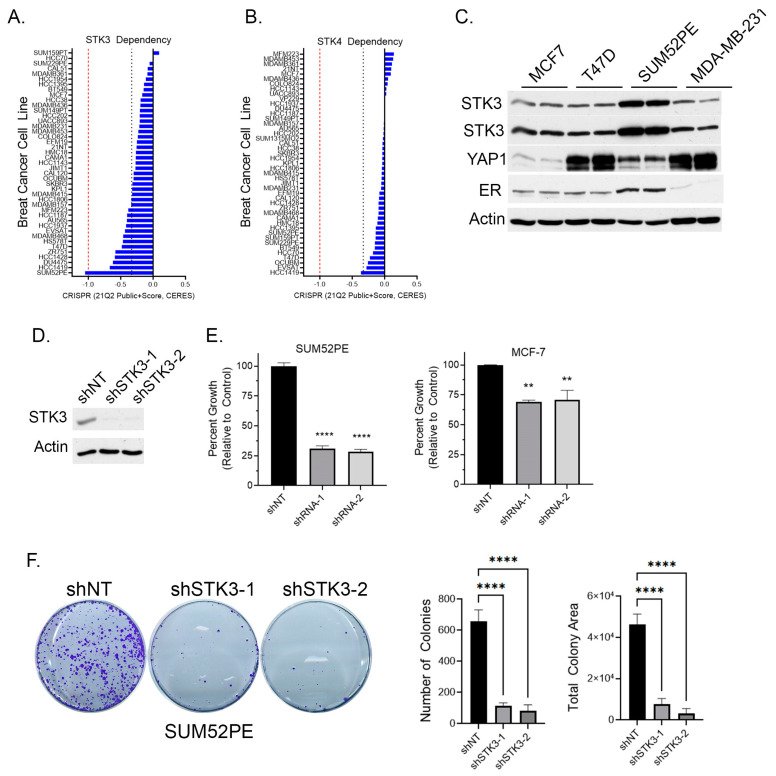
(**A**) BCa cell dependency of Hippo kinase genes STK3 and (**B**) STK4 (DepMap). Red dotted line is score of pan-essential genes. Black dotted line is CERES score of -0.33. (**C**) Western blot analysis of denoted BCa cell lines for STK3. (**D**) Validation of STK3 knock down by two shRNAs in SUM52PE cells. (**E**) Percent growth of SUM52PE and MCF-7 cell lines expressing non-targeting control or shRNAs targeting STK3. One-way ANOVA, *n* = 4, **** *p* < 0.0001, ** *p* < 0.01. (**F**) Representative images of colonies after 2 weeks of SUM52PE cells with denoted shRNAs. Quantification of colonies number and total colony area using Image J. One-way ANOVA, *n* = 6, **** *p* < 0.0001. Uncropped WB images were shown in Figure S1.

To determine if BCa cell lines are dependent on STK3, we queried the DepMap webportal for STK3 and STK4 gene dependency scores in BCa cells based on the CRISPR proliferation screen CERES score [33]. A lower CERES score indicates a higher likelihood that the gene of interest is essential in the queried cell line, a score closer to 0 indicates the gene is not essential, and −1 is comparable to the median of all pan-essential genes (red dotted line in figure) [33]. Several BCa cell lines, of varying subtypes, are in part dependent on STK3, but not STK4 (Figure 2A,B). The SUM52PE (ER+ Her2+) cell line was found to be the most dependent on STK3 expression (Figure 2A). Overall, STK3 dependency varies across BCa cells lines and cell lines are much more STK3 dependent compared to STK4 (Figure 2B).

Relative to other BCa cell lines, the Western blot analysis shows elevated STK3 expression in SUM52PE cells (Figure 2C). The protein levels of the STK3 downstream target YAP varied dramatically amongst the four cell lines. In cell proliferation assays, STK3 depletion with two different STK3-targeting shRNAs (Figure 2D) resulted in the growth inhibition of SUM52PE cells by approximately 75% (Figure 2E) and MCF-7 cells by ~25%. The loss of STK3 also significantly inhibited the capacity of SUM52PE cells to form colonies (Figure 2F).

### 3.2. STK3i Compounds Inhibit BCa Cell Proliferation and Colony Formation

**Figure 3:**
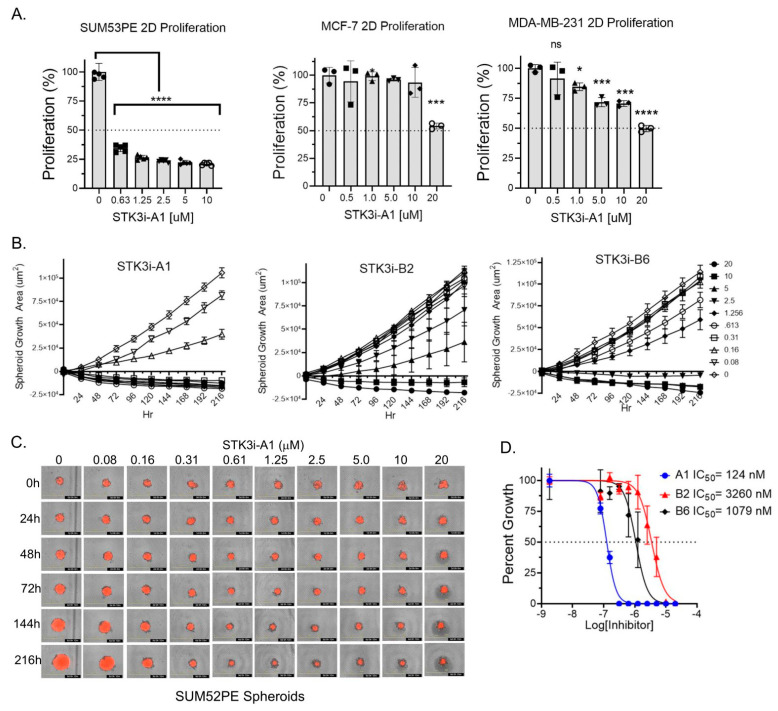
(**A**) Percent growth of SUM52PE, MDA-MB-231, and MCF-7 cell lines in standard 2D 96-well plates after 96 h of treatment with STK3i-A1. One-way ANOVA and Dunnett’s multiple comparison test, * *p* < 0.05, *** *p* < 0.001, **** *p* < 0.0001. (**B**) Longitudinal SUM52PE tumor spheroid growth treated with denoted STK3i compounds and (**C**) representative brightfield and RFP images of SUM52PE spheroids treated with increasing doses of STK3i-A1 (*n* = 5) in 384-well ULA plate. (**D**) Dose–response curve fit at day 9 of treatment. STK3i-A1 r^2^ = 0.97, STK3i-B2 r^2^ = 0.81, STK3i-B6 r^2^ = 0.92.

To determine if STK3 is a druggable target in BCa, we utilized compounds we recently characterized and validated as STK3 inhibitors with low off-target activity [13]. In standard proliferation assays, SUM52PE cells were dramatically growth-inhibited by STK3i-A1. In contrast, a moderate but dose-dependent response was observed in MDA-MB-231 (TNBC) and MCF-7 (luminal A) cells treated with STK3i-A1 (Figure 3A). We next tested three STK3i compounds on SUM52PE tumor spheroid growth. As shown in Figure 3B–D, SUM52PE spheroids were growth-inhibited in a dose-dependent manner by all three STK3i compounds. STK3i-A1 had the lowest IC50 at 124 nM (Figure 3D).

### 3.3. STK3i Compounds Inhibit BCa Colonization and Metastatic Potential

**Figure 4:**
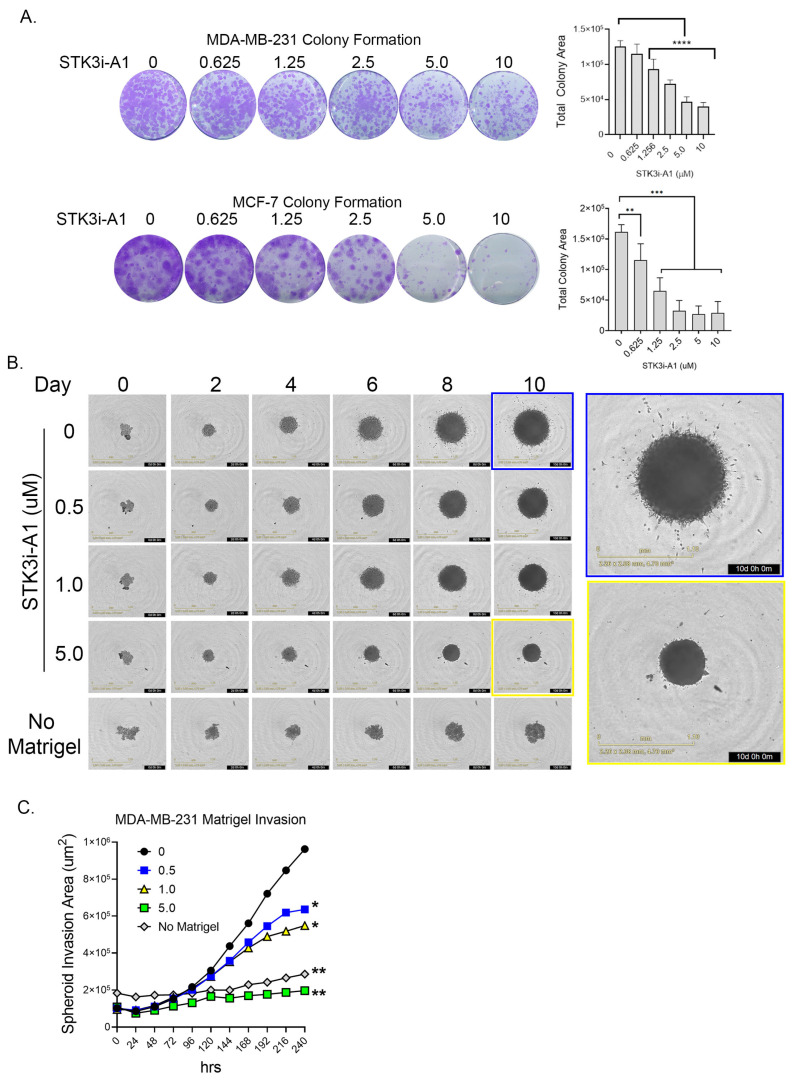
(**A**) Representative images of colony formation assay of MDA-MB-231 and MCF-7 after 14 days treated as denoted with STK3i-A1. Bar graphs represent total colony area, (*n* = 6). One-way ANOVA with Dunnett’s multiple comparisons test, ** *p* < 0.01, *** *p* < 0.001, **** *p* < 0.0001. (**B**) Representative brightfield images MDA-MB-231 spheroid invasion in matrigel at denoted days. (**C**) Longitudinal analysis of spheroid invasion area, 2-way repeated measures ANOVA with Dunnett’s multiple comparisons test, (*n* = 4). * *p* = 0.01, ** *p* < 0.001.

In colony formation assays compared to standard proliferation, a more dramatic response was observed in both the TNBC cell line MDA-MB-231 and the ER+ luminal A cell line MCF-7 upon STK3i-A1 treatment (Figure 4A). In a 3D MDA-MB-231 tumor spheroid invasion assay, treatment with STK3i-A1 significantly decreased the 3D matrigel invasion at all tested doses (Figure 4B,C).

### 3.4. STK3i Antagonizes Effects of Doxorubicin in H9C2 Cardiomyocytes

**Figure 5:**
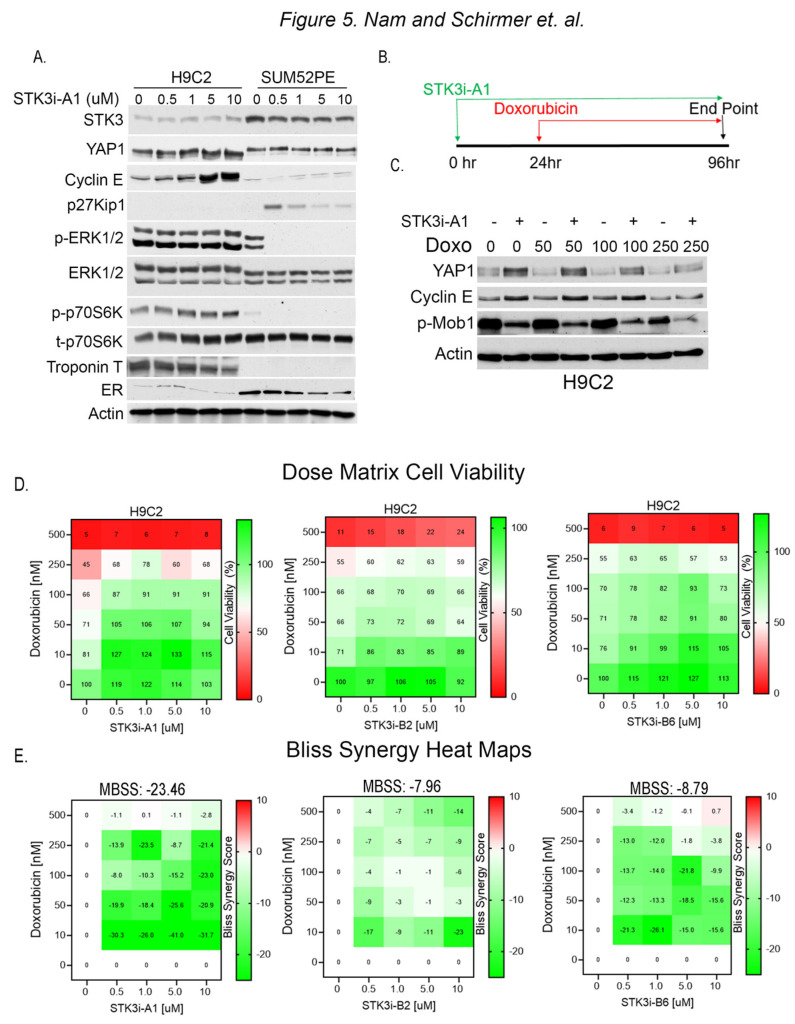
(**A**) Western blot analysis of lysates from H9C2 and SUM52PE cells treated with denoted doses of STK3i-A1 in H9C2 and SUM52PE cells for 48 h. Troponin T is used as a marker of cardiac cells and ER is used as a marker of BCa cells. (**B**) Treatment scheme for STK3i cardioprotection cell-based assays. (**C**) Western blot analysis of H9C2 cells pretreated with STK3i-A1 5 µM and then varying doses of DOX. (**D**) STK3i-DOX dose matrix H9C2 cell viability (%) dose response heatmap, each treatment *n* = 2, experiment repeated 3 times. (**E**) Individual Bliss synergy scores of the various STK3i-DOX combinations, MBSS = mean Bliss synergy score. Uncropped WB images were shown in Figure S1.

To test for divergent roles of STK3 in BCa and cardiomyocytes, we evaluated the response to STK3i-A1 in H9C2 rat cardiomyocytes and SUM52PE cells side by side. The Western blot analysis of lysates from H9C2 and SUM52PE cells treated for 48 h shows STK3 inhibition results in contrasting effects (Figure 5A). In the H9C2 cells, the total YAP levels are increased, consistent with STK3 inhibition, while in the SUM52PE cells there is no change in YAP. Further, cell cycle progression marker Cyclin E1 is induced in H9C2 cells. In contrast, SUM52PE cells upon STK3 inhibition show an induction of cell cycle inhibitor p27Kip1 and a decrease in pERK1/2, a fundamental activated pathway for cancer development and progression. These results demonstrate that STK3 inhibition in these two tissue types leads to dramatically different cellular signaling responses.

To assess the potential of STK3i-A1 as a prophylactic agent to mitigate DOX-mediated cardiotoxicity, we pretreated H9C2 cells with the compound for 24 h, followed by varying doses of DOX for 72 h (Figure 5B). The Western blot analysis of the H9C2 cells shows that STK3/4 inhibition causes the stabilization of YAP levels and the upregulation of Cyclin E1 levels (Figure 5C). In contrast, p-MOB1 levels are reduced due to the inhibition of STK3/4 kinase activity.

We next conducted dose matrix combination cell viability studies with STK3i compounds and DOX. The H9C2 cells were pretreated, as shown in Figure 5B, with STK3i-A1, STK3i-B2, and STK3i-B6 for 24 h, followed by doxorubicin for 72 h, and the cell viability was assessed after a total of 96 h. All the STK3i compounds increased the viability of the H9C2 cells at various dose combinations, as assessed by the individual Bliss synergy scores (Figure 5E). However, STK3i-A1 showed the strongest antagonistic response to DOX with an overall mean Bliss synergy score of −23.46, which indicates a synergistically antagonistic effect in combination with DOX.

### 3.5. STK3i Compounds Provide Additive BCa Growth Inhibitory Effects with Combined with Doxorubicin

**Figure 6:**
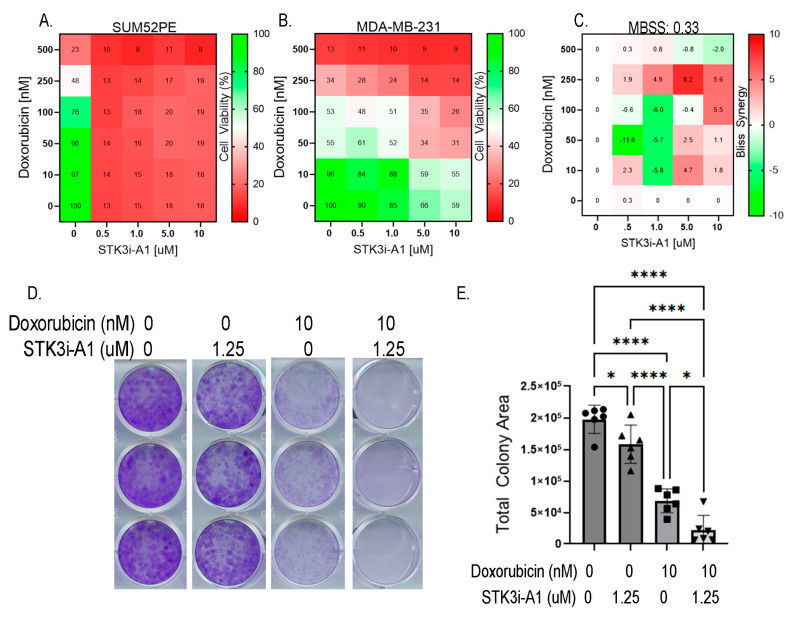
(**A**) STK3i-A1-DOX combination matrix viability dose response in SUM52PE and (**B**) MDA-MB-231 cells. BCa cells pretreated with STK3i-A1 for 24 h, then treated with DOX for 72 h as described in 5B. (**C**) Bliss synergy scores for MDA-MB-231 STK3i-A1-DOX combination. (**D**) Representative images of crystal violet-stained colony assays. (**E**) Quantified colony area of MDA-MB-231 after 14 days treated with STK3i-A1, DOX, or the combination. One-way ANOVA with Tukey’s multiple comparisons test (*n* = 6). * *p* < 0.05, **** *p* < 0.0001.

Lastly, we tested whether STK3i compounds would interfere with DOX BCa therapy. We then conducted the same dose combination studies using doxorubicin and STK3i but in BCa cell lines. The SUM52PE cells were particularly sensitive to the STK3i-A1 treatment, reducing viability to 13% of the control at the lowest dose tested (0.5 μM) so synergy was not able to be seen (Figure 6A). Importantly, STK3i did not interfere with the DOX treatment, with a mean Bliss synergy score near 0 (Figure 6C), and in fact, the STK3i-A1 compounds had moderate additive growth inhibitory effects when combined with DOX on MDA-MB-231 cell proliferation (Figure 6B). We also performed combination studies using colony formation assays and found that there was an additive growth inhibitory effect with STK3i and doxorubicin in the MDA-MB-231 cell line (Figure 6D,E). These results suggest that even in BCa that is not STK3 driven, STK3 inhibition will not interfere with DOX chemotherapy.

## 4. Discussion

Our studies reinforce the notion that STK3 plays a noncanonical role in BCa [13]. While these findings contradict the current signaling dogma that STK3 is a tumor suppressor, it is not an implausible notion. In fact, we have recently published that STK3 is frequently amplified in prostate cancer and has a noncanonical prostate cancer supportive role [13]. Moreover, there are emerging studies that suggest that STK3 and other Hippo kinases have noncanonical roles in certain cancer types. In this regard, a subset of acute myeloid leukemias were also found to be dependent on STK3 signaling, and targeting STK3 in these subsets was beneficial [14]. In STK3-dependent leukemia, it was proposed that STK3 regulates cycle-dependent kinase 1 (CDK1) to drive cell proliferation [14]. Likewise, Hippo kinases LATS1 and LATS2, the immediate downstream phospho-targets of STK3/4, were also shown to be essential for tumor cell growth in a colon cancer [34]. As another example, STK3 is overexpressed in gastric cancer, and its abundance predicts unfavorable clinical outcomes by driving cell cycle progression by activating the Ras-MAPK signaling pathway [15]. Most pertinent to this proposal, Park et al. reported that in ER^+^ BCa, the loss of STK3 (also known as MST2) induced apoptosis in vitro and slowed MCF-7 tumor growth in vivo [12]. Taken as a whole, these reports reinforce the relevance of a noncanonical role of STK3 in several cancers, including BCa.

Our genetic and pharmacological studies show that STK3 inhibition suppresses BCa proliferation, colony formation, and matrigel invasion. The response to STK3 inhibition varied by the cell BCa line. SUM52PE, the cell line with the highest endogenous levels of STK3 and the highest dependence of STK3 of the BCa cell lines tested in this study, accordingly, showed the greatest decrease in proliferation and colony formation. However, other cell lines such as MCF-7 (ER+) and MDA-MB-231 (TNBC), while resistant to STK3i in standard proliferation assays, were dramatically inhibited by STK3i in colony formation assays and 3D matrigel invasion. Further, recent animal studies have indicated that anthracycline-based chemotherapy, while effective at shrinking the primary tumor size, exacerbates the properties of BCa that encourage cell escape throughout the bloodstream and metastasis [35,36]. Thus, STK3 inhibition which slows invasion and metastasis may be a particularly beneficial therapy for BCa patients undergoing chemotherapy. Overall, our data suggest that some BCa may be more dependent on STK3 and may have increased benefit from STK3 inhibition compared to others. Importantly, STK3 loss or inhibition in BCa cells that are not dependent on STK3 did not induce cell growth and proliferation.

The widespread use of anthracycline-based chemotherapy has contributed to the increased risk of cardiomyopathy in BCa patients [18]. In this study, we examined the potential of STK3 as a target to induce cardioprotection while also facilitating the antitumor effects of anthracyclines. Consistent with previous reports, Western blot and cell viability assay analyses of H9C2 cells treated with DOX showed that DOX induces apoptosis in H9C2 by degrading YAP1 [26,37]. Pretreatment with our STK3i compound both increased the baseline YAP1 levels and reversed DOX-induced YAP1 degradation. The Bliss synergy analysis of the H9C2 cells treated with a dose combination matrix showed that STK3i-A1 restored cell viability and antagonized the inhibitory effects of DOX. Previous studies have proposed that YAP1 is an essential mediator of cell cycle entry into the S-phase by upregulating cell cycle regulators [38]. Accordingly, we have found that STK3 inhibition induces Cyclin E1 expression even in the presence of doxorubicin. This suggests that the cardioprotective effect of STK3 inhibitors observed here is the result of increased cardiomyocyte proliferation through the upregulation of cell cycle progression. Although previous studies have produced similar results by directly amplifying YAP1 activity in H9C2 cells [26,27], we have shown here that YAP1-mediated cardioprotection can be accomplished by inhibiting the upstream kinases STK3/4, which is important because these enzymes have stronger translational potential as druggable targets than YAP1 [29]. While these results are promising, we only tested the effect of STK3 inhibition on an H9C2 immortalized rat cardiomyocyte cell line. Further studies in improved cardiac models such as primary or engineered hIPSC cardiomyocytes are needed, along with in vivo pre-clinical cardioprotection mouse studies.

We tested if inhibition using STK3i would counteract or interfere with the therapeutic effects of DOX on BCa cells. As evidenced by the Bliss synergy analysis and colony formation assays, pharmacological STK3 inhibition did not counteract the doxorubicin-induced decrease in BCa cell viability, but rather enhanced it. This is especially interesting given that in our patient survival data, high STK3 expression was correlated with lower rates of survival in patients receiving chemotherapy. This suggests that STK3 may be a particularly appealing target for these patients with high expression because STK3i can have an added effect with the chemotherapy they are receiving. Contrary to its effect in H9C2 cells, STK3 inhibition had no effect on the YAP1 and Cyclin E1 levels in BCa. STK3i inhibition in BCa increased the levels of cell cycle inhibitor p27Kip1, slowing cell cycle progression, and decreased p-ERK1/2, an essential signaling pathway for cancer progression.

## 5. Conclusions

Our studies provide further evidence that contradicts the previously accepted role of STK3 as a universal tumor suppressor and that in some cancers it has a tumor supportive role. The mechanism of action behind the noncanonical role of STK3 in BCa remains to be elucidated. As a tumor suppressor in normal tissues such as the heart, our studies suggest that we may take advantage of STK3 inhibition to prime normal cells for protection against anthracycline chemotherapy. Beyond BCa, the development of STK3 inhibitors to mitigate the effects of chemotherapy may have a broad impact, such as in childhood cancer survivors who are seven times more likely to die prematurely from cardiac disease than the general population [39]. Taking advantage of the druggable divergent roles of STK3 in BCa and normal cells may lead to an increase in overall cancer patient survivorship.

## Data Availability

The data that support the findings of this manuscript are available upon request from the corresponding author.

## Supplementary Materials

The following supporting information can be downloaded at: https://www.mdpi.com/article/10.3390/cancers15102817/s1, Figure S1: uncropped WB images.
