# Supplementary material for: Targeting the Divergent Roles of STK3 Inhibits Breast Cancer Cell Growth and Opposes Doxorubicin-Induced Cardiotoxicity In Vitro
Source: Cancers (Basel). 2023 May 18;15(10):2817. doi: 10.3390/cancers15102817 (PMC10216518; doi:10.3390/cancers15102817)

Figure 2C

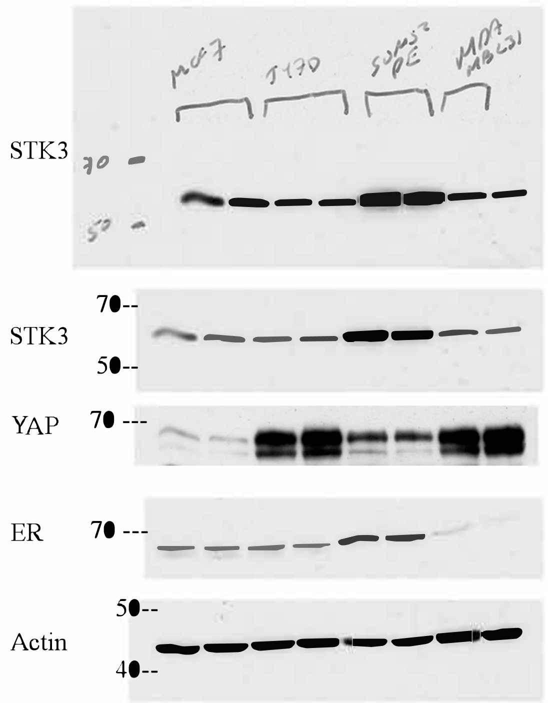

Figure 2D

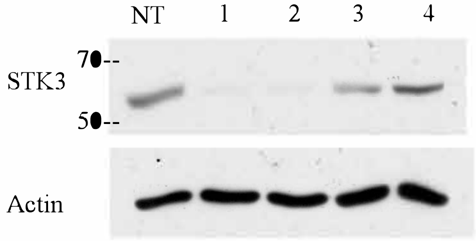

Four STK3 shRNAs tested,
shRNA 1 and shRNA-2 used for studies

Figure 5A

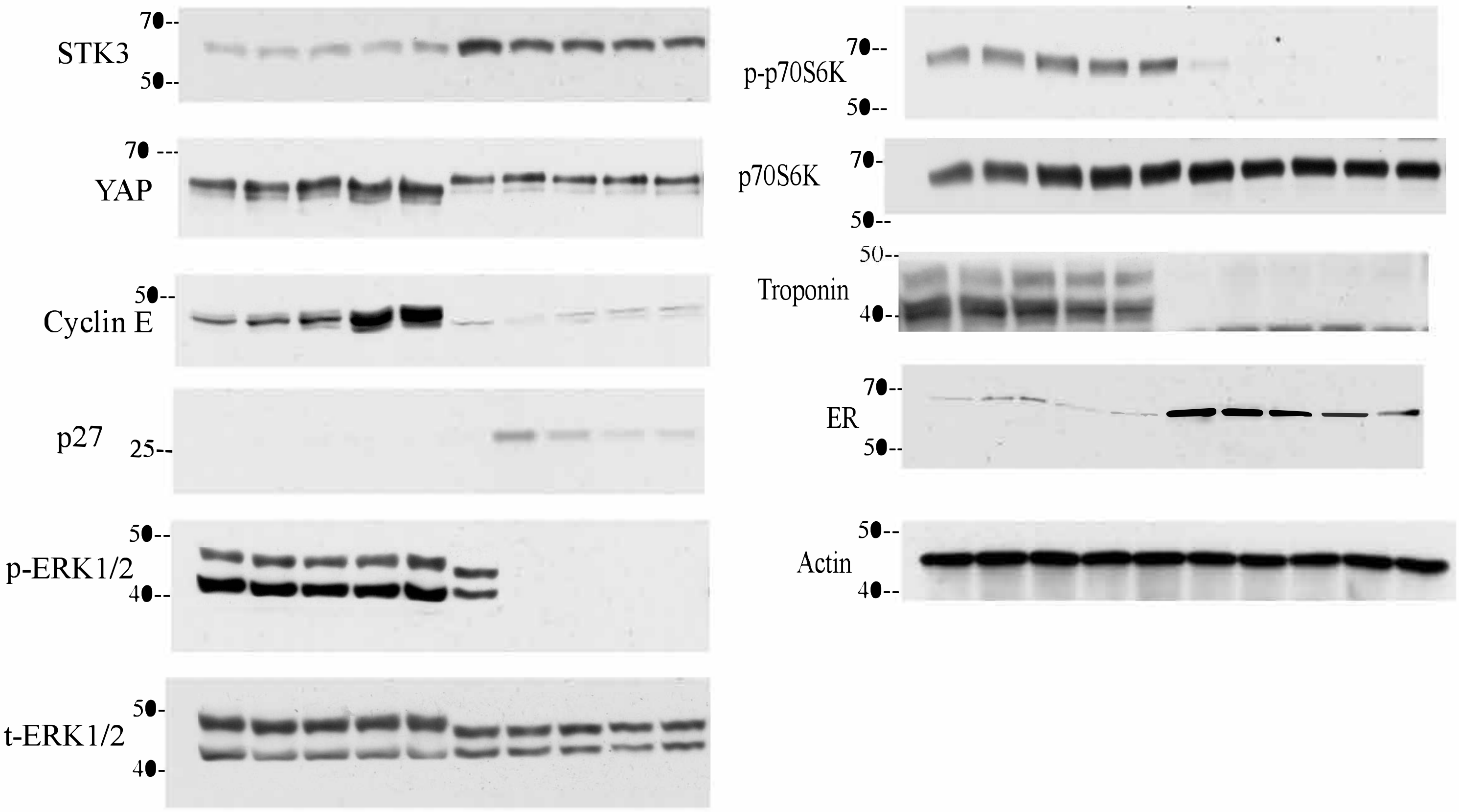

Figure 5C

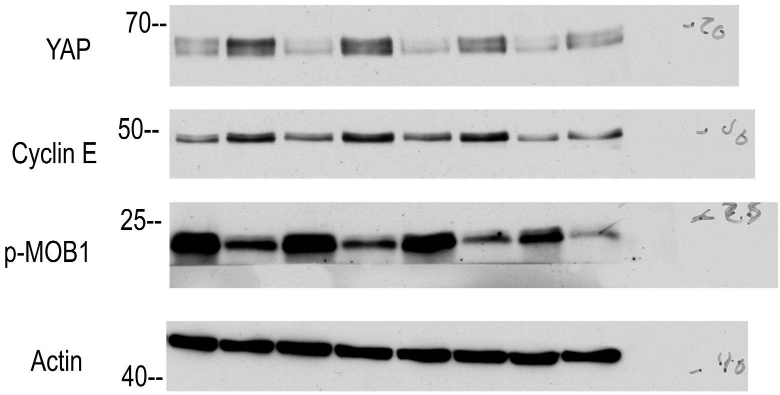

Western Blot Densitometry

Figure 2C Densiometry

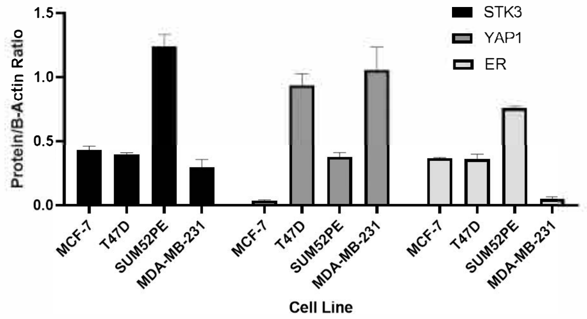

Fig. 2D Densiometry

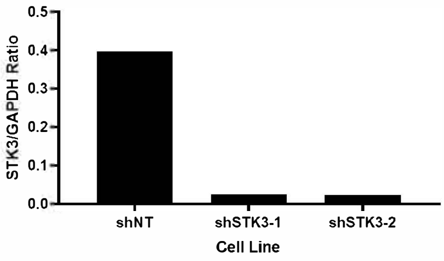

Figure 5A Densiometry - H9C2

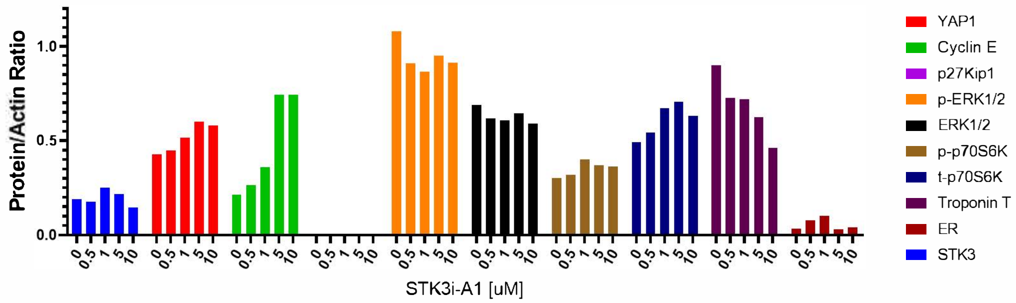

Figure 5A Densiometry - SUM52PE

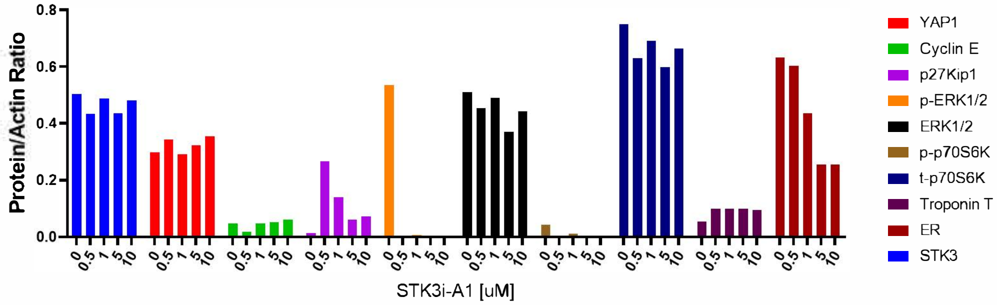

Figure 5C Densiometry

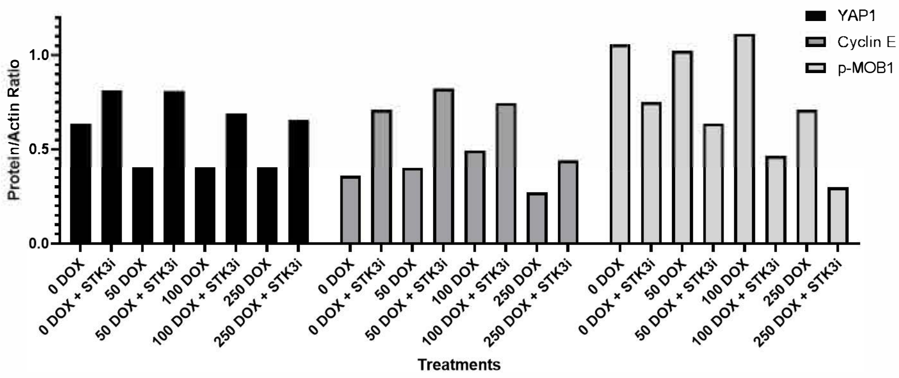

Supplement: Supplementary file 1 [file cancers-15-02817-s001.zip › cancers-2321524-supplementary.pdf]
